# Association of soluble ST2 with all-cause and cardiovascular mortality in renal transplant recipients: a single-centre cohort study

**DOI:** 10.1186/s12882-020-1690-6

**Published:** 2020-01-28

**Authors:** Paul A. Devine, Christopher Cardwell, Alexander P. Maxwell

**Affiliations:** 1 Regional Nephrology and Transplant Unit, Belfast City Hospital, Belfast, UK; 2 Centre for Public Health, Queen’s University Belfast, Belfast, UK

**Keywords:** Kidney transplantation, Cardiovascular diseases, Biomarkers, Risk factors, C-reactive protein

## Abstract

**Background:**

Soluble ST2 is a novel biomarker of myocardial fibrosis with an established role in prognostication of patients with heart failure. Its role in cardiovascular risk prediction for renal transplant recipients has not been investigated despite promising results for ST2 in other populations with renal disease.

**Methods:**

In this prospective cohort study, 367 renal transplant recipients were followed up for a median of 16.2 years to investigate the association of soluble ST2 concentration with all-cause mortality. Cardiovascular mortality and major adverse cardiovascular events were secondary outcomes. Cox regression models were used to calculate hazard ratios and 95% confidence intervals for ST2 before and after adjustments. ST2 concentration was analysed both as a continuous variable and following categorisation according to the recommended cut-point of 35 ng/ml.

**Results:**

A twofold higher ST2 concentration was associated with a 36% increased risk of all-cause mortality after adjustment for conventional cardiovascular risk factors and high-sensitivity C-reactive protein (adjusted hazard ratio 1.36; 95% confidence interval 1.06–1.75; *p* = 0.016). Associations with ST2 concentration were similar for cardiovascular events (adjusted hazard ratio 1.31; 95% confidence interval 1.00–1.73; *p* = 0.054), but were stronger for cardiovascular mortality (adjusted hazard ratio 1.61; 95% confidence interval 1.07–2.41; *p* = 0.022). Addition of ST2 to risk prediction models for mortality and cardiovascular events failed to improve their predictive accuracy.

**Conclusions:**

ST2 is associated with, but does not improve prediction of, adverse outcomes in renal transplant recipients.

## Background

Transplantation has been established as the optimal treatment for end-stage renal disease (ESRD). It substantially improves survival compared to dialysis [1]. However, life expectancy in renal transplant recipients (RTR) is lower than in their age-matched peers [2]. As the leading cause of death following kidney transplantation, reducing cardiovascular disease remains an important goal in improving overall patient survival [2].

Traditional cardiovascular risk factors are prevalent in patients with ESRD both before and after transplantation [3]. Post-transplantation, risk factors such as dyslipidaemia are exacerbated by the immunosuppressant medication necessary to reduce immunological injury to the allograft [4]. Additional factors including impaired graft function and proteinuria also contribute to this excess cardiovascular risk [3]. Because of this unique combination of factors, the clinical presentation of cardiovascular disease may differ to that of the general population. Non-atherosclerotic abnormalities such as myocardial fibrosis and left ventricular hypertrophy are common in ESRD [5]. Consequently, over half of cardiovascular-related deaths in RTR are due to arrhythmias or cardiac arrest [2]. It is therefore unsurprising that risk scores used for the general population underestimate risk of cardiovascular events and mortality when applied to RTR [6]. A cardiovascular risk calculator has been specifically designed for use in RTR [7, 8]. While it outperforms other scores in this patient cohort, there may be scope to further improve its accuracy [6].

In cardiovascular medicine, recent emphasis has been placed on using biomarkers to aid prediction of adverse outcomes [9]. Soluble ST2 (sST2) is a member of the IL-1 receptor family which is measurable in human plasma [10]. sST2 expression is upregulated by myocardial stress, and this has been linked to cardiac hypertrophy and fibrosis [11]. Elevated sST2 concentration predicts mortality in patients with heart failure and stable ischaemic heart disease [12, 13]. This appears to be independent of renal function [14]. Recent studies have also reported sST2 to be of prognostic value in patients with chronic kidney disease (CKD) and ESRD requiring haemodiafiltration [15, 16].

To our knowledge, there are no data on the utility of sST2 as a prognostic biomarker in RTR. In this study, we investigated the association of sST2 with mortality and major adverse cardiovascular events (MACE) in RTR. We also evaluated whether adding sST2 to survival models comprised of established risk factors and high-sensitivity C-reactive protein (hs-CRP) improved the prognostic accuracy of such models in this population.

## Materials and methods

### Study population

From June 2000 until December 2002, 379 renal transplant recipients in Northern Ireland were recruited from outpatient clinics at Antrim Area Hospital and Belfast City Hospital. All recipients with a functioning graft (i.e. independent of dialysis at time of recruitment) were eligible for inclusion. No formal exclusion criteria were imposed. Written consent was obtained from all participants. This study was performed in accordance with the Declaration of Helsinki. A favourable ethical opinion was obtained from an NHS Research Ethics Committee (17/LO/1799).

At recruitment, a brief physical assessment was performed. Body mass index (BMI) was calculated. Blood pressure was measured at three consecutive clinic visits and the mean value recorded. The presence of left ventricular hypertrophy (LVH) on electrocardiogram (ECG), according to Sokolow-Lyon voltage criteria, was documented. Participants completed a 24-h urine collection to quantify proteinuria. A fasting blood sample was drawn from each participant and separated into aliquots of serum and plasma. Routine biochemical and haematological analyses were performed by NHS laboratories on the day of recruitment.

Face-to-face questionnaires and a review of medical notes were undertaken to determine baseline demographic data, cause and duration of ESRD, details of the transplant procedure, co-morbidities and pre-existing cardiovascular disease. Medications, including immunosuppression regimen, were also recorded.

### Biomarker measurement

sST2 concentration was measured once for each participant. This was performed using EDTA-plasma samples which had been collected on the day of study recruitment (ranging June 2000 – December 2002). Plasma samples had been stored at − 80 °C from the day of study recruitment until analysis in September 2018. All were first-thaw samples. All analyses were performed in the same laboratory.

The process was fully-automated using a Triturus analyser (Diagnostics Grifols SA, Barcelona, Spain) and the Presage® ST2 assay (Critical Diagnostics, San Diego, CA, USA). This high-sensitivity, enzyme-linked immunoassay (ELISA) has a lower limit of detection of 2 ng/ml, with a reportable range of 3.1–200.0 ng/ml. All samples were analysed in duplicate and measurement was repeated for any sample with a coefficient of variation (CV) > 10%. Absorbances were measured using spectrophotometry at 450 nm, and sST2 concentration determined from a log-log linear regression curve. The intra-assay CV was < 2%. The inter-assay CV was 4.1% at 30.4 ng/ml and 5.1% at 74.8 ng/ml.

The concentration of hs-CRP was determined from serum samples stored and treated in an identical manner to the plasma samples. The CRPL3 assay and a Cobas® 8000 modular analyser (Roche Diagnostics, Burgess Hill, UK) were used. The measurement range of the assay is 0.3–350 mg/L. The intra-assay CV was < 2%. The inter-assay CV was 2.16% at 15 mg/L and 2.70% at 129 mg/L.

### Outcomes and follow-up

Prospective follow-up data on participants were obtained from the Northern Ireland Kidney Transplant Database (Ethics Committee reference: 18/NI/0004). This database prospectively records outcomes for all kidney transplant procedures performed in Northern Ireland, including recipient and graft survival and the incidence of complications.

The primary outcome was time to all-cause mortality. Secondary outcomes were time to cardiovascular mortality and time to first MACE. MACE was defined as myocardial infarction (based on two of the following three: history, typical ECG changes, troponin rise), ischaemic heart disease requiring coronary artery stenting or bypass grafting, congestive cardiac failure requiring hospitalisation, pulmonary embolism, stroke (diagnosed clinically or radiologically), and peripheral vascular disease requiring radiological intervention or amputation.

### Statistical analyses

Categorical variables are presented as counts and percentages. Continuous variables are presented as mean and standard deviation (SD) or median and interquartile range (IQR) as appropriate to their distribution. The chi-square test, Student’s t-test, Mann-Whitney U test or Kruskal-Wallis test were used to compare differences between groups. Spearman’s rank correlation coefficient was used to investigate the relationship between laboratory parameters and sST2 concentration.

Kaplan-Meier curves were plotted to demonstrate the survival experience by group. The log-rank test was employed to investigate differences in survival between groups. Hazard ratios (HR) and 95% confidence intervals (CI) were obtained from Cox proportional hazards regression analyses. In survival models, sST2 was treated as a continuous predictor variable following logarithmic transformation (to base 2). sST2 concentration was also categorised according to the widely reported cut-off value of 35 ng/ml.

Additionally, logistic regression analysis was performed with recipient survival outcomes at 15 years as the dependent variable. Youden’s J statistics were subsequently calculated from receiver operator curve (ROC) analyses to determine the optimal cut-point for sST2 concentration with the highest sensitivity and specificity in this cohort [17]. Further survival analyses were undertaken using these optimal cut-off values to categorise sST2.

Covariates included in survival models were selected a priori based upon their reported role as cardiovascular risk factors in the existing literature. The covariates in Model 1 were adopted (as far as possible from the available baseline data on study participants) from the QRISK2 score [18]. This is a cardiovascular risk prediction tool recommended in national guidelines for use in the general population of the UK [19]. Additional covariates relevant to renal transplant recipients (eGFR, proteinuria and hs-CRP) were also included.

The covariates in Model 2 were adopted from the Cardiovascular Risk Calculator for Renal Transplant Recipients. This risk calculator was derived from a cohort of renal transplant recipients in the Assessment of Lescol in Renal Transplantation (ALERT) trial and has been externally validated [7, 8]. Two versions of this calculator exist; one for the prediction of mortality (covariates included in Model 2a) and one for the prediction of MACE (covariates included in Model 2b).

The impact of sST2 on the predictive accuracy of each survival model was evaluated using discrimination metrics: difference in C-statistics (before and after addition of sST2), integrated discrimination improvement (IDI) and category-free net reclassification index (NRI(> 0)) [20, 21].

The statistical software package R V3.5.2 (http://www.R-project.org) was employed to derive discrimination metrics using the ‘compareC’ and ‘survIDINRI’ packages. SPSS (Version 24) was used for all other analyses.

## Results

### Characteristics of the study population

Plasma samples were unavailable for 12 participants due to insufficient volume at the time of sampling. sST2 concentration was measured for 367 of the 379 recruited patients.

Baseline characteristics are displayed in Table 1. The median age of participants was 47 years. The majority were male and non-smokers. In total, 13.6% had diabetes mellitus, 80.4% had hypertension and 21.8% had pre-existing cardiovascular disease. The median time between transplantation and sST2 measurement was 7.8 years. Mean estimated glomerular filtration rate (eGFR) was 52.4 ml/min/1.73m^2^.

**Table 1:** Baseline characteristics of the study population

Characteristic	Total cohort *N* = 367	sST2 < 35 ng/ml *N* = 197	sST2 ≥ 35 ng/ml *N* = 170	*P* value
Age (years); median (IQR)	47 (38, 60)	44 (37, 58)	49 (38, 60)	0.138
Male sex; n (%)	234 (63.8)	113 (57.4)	121 (71.2)	0.008
Primary renal disease; n (%)				
Glomerular disease	90 (24.5)	48 (24.4)	42 (24.7)	1.000
Interstitial disease & pyelonephritis	79 (21.5)	47 (23.9)	32 (18.8)	0.297
Polycystic kidney disease	48 (13.1)	20 (10.2)	28 (16.5)	0.102
Diabetic nephropathy	23 (6.3)	12 (6.1)	11 (6.5)	1.000
Aetiology unknown	51 (13.9)	26 (13.2)	25 (14.7)	0.791
Other	76 (20.7)	44 (22.3)	32 (18.8)	0.485
Number of grafts–including current (%)				
1	314 (85.6)	170 (86.3)	144 (84.7)	0.777
2	47 (12.8)	26 (13.2)	21 (12.4)	0.932
3	5 (1.4)	1 (0.5)	4 (2.4)	0.187
4	1 (0.3)	0 (0)	1 (0.6)	0.463
Donor type; n (%)				
DBD	341 (92.9)	182 (92.4)	159 (93.5)	0.824
Living-related	26 (7.1)	15 (7.6)	11 (6.5)	
Time (months); median (IQR)				
Total RRT (dialysis + transplant)	125 (69, 193)	120 (65, 193)	129 (76, 190.3)	0.193
Post-transplant	94 (43, 195)	94 (42.5, 169.5)	103.5 (45, 157.5)	0.673
Immunosuppression; n (%)				
Steroid use	282 (76.8)	139 (70.6)	143 (84.1)	0.003
CNI use	246 (67.0)	130 (66.0)	116 (68.2)	0.730
Sirolimus use	11 (3.0)	5 (2.5)	6 (3.5)	0.804
Current smokers; n (%)	67 (18.3)	42 (21.3)	25 (14.7)	0.134
History of diabetes mellitus; n (%)	50 (13.6)	28 (14.2)	22 (12.9)	0.840
History of hypertension; n (%)	295 (80.4)	154 (78.2)	141 (82.9)	0.310
Statin use; n (%)	154 (42.0)	82 (41.6)	72 (42.4)	0.972
BMI (kg/m^2^); mean (SD)	26.6 (4.5)	26.7 (5.0)	26.4 (4.0)	0.721
Left ventricular hypertrophy; n (%)	71 (20.8)	38 (20.2)	33 (21.4)	0.887
History of cardiovascular disease; n (%)	80 (21.8)	34 (17.3)	46 (27.1)	0.032
Ischaemic heart disease	55 (15.0)	23 (11.7)	32 (18.8)	0.077
Stroke	20 (5.4)	9 (4.6)	11 (6.5)	0.569
Peripheral vascular disease	24 (6.5)	8 (4.1)	16 (9.4)	0.063
Total cholesterol (mmol/L); mean (SD)	5.3 (1.0)	5.3 (1.0)	5.2 (1.0)	0.371
HDL cholesterol (mmol/L); mean (SD)	1.4 (0.4)	1.4 (0.4)	1.4 (0.4)	0.866
LDL cholesterol (mmol/L); mean (SD)	3.0 (0.8)	3.0 (0.8)	3.0 (0.8)	0.340
Triglycerides (mmol/L); mean (SD)	2.0 (1.2)	2.0 (1.3)	1.9 (1.0)	0.582
Creatinine (μmol/L); mean (SD)	145.6 (69.7)	143 (67)	149 (73)	0.449
eGFR (ml/min/1.73m^2^); mean (SD)	52.4 (20.5)	52 (19)	52 (22)	0.955
CKD stage; n (%)				
1&2 (eGFR ≥60 ml/min/1.73m^2^)	117 (31.9)	68 (34.5)	49 (28.8)	0.291
3 (eGFR 30–59 ml/min/1.73m^2^)	201 (54.8)	100 (50.8)	101 (59.4)	0.120
4 (eGFR 15–29 ml/min/1.73m^2^)	42 (11.4)	25 (12.7)	17 (10.0)	0.520
5 (eGFR < 15 ml/min/1.73m^2^)	7 (1.9)	4 (2.0)	3 (1.8)	1.000
Proteinuria (mg/24 h)^a^; median (IQR)	200 (100, 500)	200 (100, 400)	200 (100, 700)	0.029
Proteinuria category^a^; n (%)				
Normal – mild (≤ 150 mg/24 h)	144 (42.0)	88 (47.8)	56 (35.2)	0.024
Moderate (151–499 mg/24 h)	113 (32.9)	60 (32.6)	53 (33.3)	0.978
Severe (≥ 500 mg/24 h)	86 (25.1)	36 (19.6)	50 (31.4)	0.016
Haemoglobin (g/dl); mean (SD)	12.8 (1.8)	12.8 (1.6)	12.7 (2.1)	0.361
HbA1c (%); mean (SD)	6.2 (1.1)	6.1 (1.2)	6.3 (1.1)	0.174
hsCRP (mg/L); median (IQR)	1.7 (0.7, 4.7)	1.6 (0.7, 4.4)	1.8 (0.7, 5.3)	0.293

*Abbreviations: BMI* body mass index, *DBD* deceased after brainstem death, *eGFR* estimated glomerular filtration rate, *HbA1c* haemoglobin A1c, *hsCRP* high-sensitivity C-reactive protein, *IQR* interquartile range, *RRT* renal replacement therapy, *sST2* soluble ST2

^a^Proteinuria data available for 343 participants

The immunosuppression regimens (not shown) were heterogeneous, representing practice in Northern Ireland in 2000–2002. Overall, 77% of participants were taking prednisolone and 67% were prescribed a calcineurin-inhibitor (CNI)-based regimen. Of those participants on a CNI-based regimen, 195 (79.3%) were using ciclosporin. Induction therapy was not used in any participant.

### Concentration of sST2 in the study population

The median sST2 concentration was 33.1 ng/ml. sST2 concentrations ranged from 9.6–177.0 ng/ml. Using the accepted cut-off of 35 ng/ml, 197 (53.7%) participants had low sST2 (< 35 ng/ml) and 170 (46.3%) participants had high sST2 (> 35 ng/ml). The baseline characteristics of participants according to low and high sST2 concentration are shown in Table 1.

Participants in the high sST2 group were more likely to be male and have a history of cardiovascular disease. There was no difference in the prevalence of LVH in the low sST2 group compared to the high sST2 group (20.2% versus 21.4%, *P* = 0.887).

In univariable analyses, there was little evidence of correlation between sST2 concentration and creatinine (Spearman’s rho 0.075, *P* = 0.153), eGFR (Spearman’s rho − 0.034, *P* = 0.521) or hsCRP (Spearman’s rho 0.065, *P* = 0.217). There was evidence of weak correlation between concentration of sST2 and proteinuria (Spearman’s rho 0.152, *P* = 0.005).

### Incidence of mortality and MACE

Follow-up data was complete for all participants. The median duration of follow-up was 16.2 years. There were 171 deaths during the study period. Cardiovascular disease was the commonest cause of mortality, accounting for 62 (36.3%) deaths in the study population. Overall, 199 MACE occurred in 131 participants.

### Association of sST2 with all-cause mortality

In an unadjusted model, the risk of all-cause mortality increased by 31% (HR 1.31; 95% CI 1.05–1.63) for every twofold increase in sST2 concentration (Table 2) and was similar after adjustments (adjusted HR 1.36; 95% CI 1.05–1.76).

**Table 2:** Association of sST2 with all-cause mortality, cardiovascular mortality and MACE in cox proportional hazards models

			Model 1		Model 2	
	Unadjusted HR (95% CI)	*P* value	Adjusted HR (95% CI)	*P* value	Adjusted HR (95% CI)	*P* value
All-cause mortality (*n* = 171)						
sST2 *(per twofold increase)*	1.31 (1.05–1.63)	0.016	1.36 (1.05–1.76)	0.018	1.33 (1.06–1.67)^a^	0.014
sST2 ≥ 35 ng/ml	1.41 (1.04–1.90)	0.025	1.45 (1.03–2.04)	0.035	1.36 (1.00–1.85)^a^	0.049
sST2 ≥ 33 ng/ml *(optimal)*	1.52 (1.12–2.05)	0.007	1.62 (1.15–2.29)	0.006	1.46 (1.07–1.99)^a^	0.016
Cardiovascular mortality (*n* = 62)						
sST2 *(per twofold increase)*	1.50 (1.05–2.13)	0.024	1.65 (1.09–2.48)	0.017	1.50 (1.03–2.18)^a^	0.033
sST2 ≥ 35 ng/ml	1.31 (0.80–2.16)	0.288	1.43 (0.80–2.56)	0.233	1.16 (0.69–1.94)^a^	0.576
sST2 ≥ 41 ng/ml *(optimal)*	1.72 (1.04–2.85)	0.035	1.90 (1.06–3.43)	0.032	1.57 (0.93–2.67)^a^	0.092
MACE (*n* = 131)						
sST2 *(per twofold increase)*	1.36 (1.07–1.74)	0.013	1.30 (0.97–1.73)	0.079	1.40 (1.08–1.80)^b^	0.010
sST2 ≥ 35 ng/ml	1.34 (0.95–1.88)	0.096	1.09 (0.74–1.63)	0.659	1.19 (0.84–1.71)^b^	0.332
sST2 ≥ 24 ng/ml *(optimal)*	1.75 (1.09–2.82)	0.021	1.70 (0.97–3.01)	0.066	1.89 (1.14–3.13)^b^	0.013

*Abbreviations: BMI* body mass index, *CI* confidence interval, *CVD* cardiovascular disease, *eGFR* estimated glomerular filtration rate, *HR* hazard ratio, *hs-CRP* high-sensitivity C-reactive protein, *IHD* ischemic heart disease, *MACE* major adverse cardiovascular events, *RRT* renal replacement therapy, *sST2* soluble ST2

Model 1: adjusted for age, sex, diabetes, history of hypertension, cholesterol/HDL ratio, BMI, smoking status, history of CVD, eGFR, proteinuria category, hs-CRP

^a^Model 2 – mortality: adjusted for age, diabetes, total RRT time (= pre-transplant dialysis time + time-post transplant), serum creatinine, smoking status, history of IHD

^b^Model 2 – MACE: adjusted for age, diabetes, LDL-cholesterol, number of transplant grafts, serum creatinine, smoking status, history of IHD

**Fig. 1:**
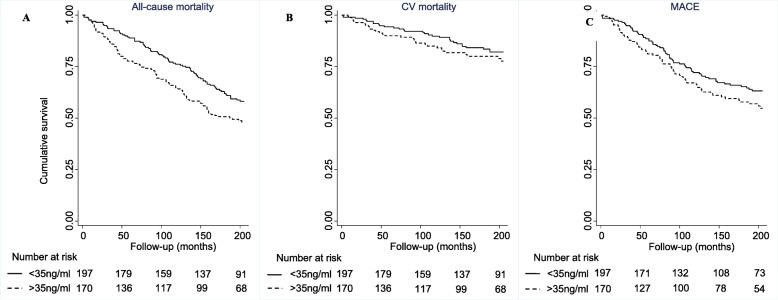
Kaplan-Meier curves of event-free survival for **a** all-cause mortality, **b** CV mortality and **c** MACE according to low (< 35 ng/ml) or high (≥35 ng/ml) sST2 concentration. Abbreviations: CV = cardiovascular; MACE = major adverse cardiovascular event; sST2 = soluble ST2

The Kaplan-Meier plot graphically shows that the survival probability was significantly greater in the low sST2 group compared to the high sST2 group (Log rank test: *P* = 0.025) (Fig. 1a). In both univariable and multivariable models, high sST2 concentration (> 35 ng/ml) was significantly associated with all-cause mortality (Model 1: adjusted HR 1.45, 95% CI 1.03–2.04; Model 2: adjusted HR 1.36, 95% CI 1.00–1.85).

Despite a significant association between sST2 concentration and all-cause mortality, the addition of sST2 as a continuous variable (per twofold increase) to survival models did not significantly improve their discrimination metrics (Table 3).

**Table 3:** Metrics for improvement in risk prediction of outcomes with addition of (continuous) sST2

	All-cause mortality	Cardiovascular mortality	MACE
Model 1			
Adjusted HR per twofold increase sST2 (95% CI)	1.36 (1.05, 1.76)	1.65 (1.09, 2.48)	1.30 (0.97, 1.73)
C-statistic			
Original model	0.767	0.809	0.773
Original model + sST2	0.770	0.809	0.772
Difference	0.003	0.000	−0.001
*P* value	0.510	0.993	0.870
IDI^a^ (95% CI)	0.004 (−0.005–0.026)	0.020 (− 0.002–0.062)	0.007 (− 0.003–0.029)
*P* value	0.472	0.096	0.269
NRI(> 0)^a^ (95% CI)	0.172 (− 0.248–0.398)	0.186 (− 0.292–0.518)	−0.014 (− 0.348–0.320)
*P* value	0.365	0.429	1.000
Model 2			
Adjusted HR per twofold increase sST2 (95% CI)	1.33 (1.06–1.67)	1.50 (1.03–2.18)	1.40 (1.08–1.80)
C-statistic			
Original model	0.757	0.792	0.756
Original model + sST2	0.761	0.791	0.756
Difference	0.004	−0.001	0.000
*P* value	0.257	0.894	0.909
IDI ^a^ (95% CI)	0.005 (−0.004–0.025)	0.014 (− 0.002–0.053)	0.011 (− 0.003–0.032)
*P* value	0.385	0.106	0.126
NRI(> 0) ^a^ (95% CI)	0.212 (− 0.128–0.432)	0.122 (− 0.188–0.428)	0.186 (−0.144–0.454)
*P* value	0.159	0.332	0.173

*Abbreviations: CI* confidence interval, *IDI* Integrated Discrimination Index, *MACE* major adverse cardiovascular events, NRI(> 0) = category-free Net Reclassification Index, *sST2* soluble ST2

^a^Based upon events up to 185 months

### Association of sST2 with cardiovascular mortality

A plot of sST2 concentration versus time post-transplant for participants who experienced cardiovascular mortality and those who did not is demonstrated in Additional file 1: Figure S1.

The unadjusted risk of cardiovascular mortality increased by 50% (95% CI 5–113%) per twofold increase in sST2 concentration (Table 2). This association with cardiovascular mortality remained significant in multivariable models (Model 1: adjusted HR 1.65, 95% CI 1.09–2.48; Model 2: adjusted HR 1.50, 95% CI 1.03–2.18). However, the C-statistics of these models were not significantly altered by the addition of sST2 concentration (Table 3).

The Kaplan-Meier plot demonstrated less marked differences in cardiovascular mortality between the low sST2 group and the high sST2 group (Log rank test: *P* = 0.286) (Fig. 1b). When categorised according to the widely accepted cut-off value of 35 ng/ml, sST2 concentration was not significantly associated with cardiovascular mortality (Table 2).

### Association of sST2 with MACE

For every twofold increase in sST2 concentration, the unadjusted risk of developing MACE increased by 36% (95% CI 7–74%) (Table 2). In Model 1, the adjusted risk of developing MACE increased 30% (95% CI 0.97–1.73) per twofold increase in sST2 concentration. A similar increase in risk was demonstrated after adjustment for the covariates in Model 2.

When categorised according to the cut-off of 35 ng/ml, sST2 concentration was not associated with development of MACE on Kaplan-Meier (Log-rank test: *P* = 0.212) (Fig. 1c), or univariable Cox survival analyses (unadjusted HR 1.34, 95% CI 0.95–1.88) (Table 2). High sST2 concentration (> 35 ng/ml) was not associated with MACE on multivariable analyses.

The association of sST2 concentration with mortality and MACE in a sex-stratified analysis is demonstrated in Additional file 1: Table S1.

### Association of Optimal sST2 concentration cut-offs with patient outcomes

Calculation of Youden’s J statistics allowed identification of the optimal cut-off values of sST2 concentration for predicting each adverse outcome in the study population. The optimal cut-off value of sST2 concentration for all-cause mortality was 33 ng/ml, which was close to the median value. An sST2 concentration greater than 33 ng/ml was associated with an increased risk of all-cause mortality in all univariable and multivariable analyses.

The optimal cut-off values for cardiovascular mortality and MACE were 41 ng/ml and 24 ng/ml respectively. When categorised according to the optimal cut-off value of 41 ng/ml, sST2 was associated with cardiovascular mortality in an unadjusted analysis (unadjusted HR 1.72, 95% CI 1.04–2.85). This relationship was attenuated following adjustment for the covariates in Model 2.

The relationship between each adverse outcome and sST2 concentration dichotomised at these cut-off values are demonstrated in Table 2. An sST2 concentration greater than 24 ng/ml was associated with a 75% increase in the risk of developing MACE (unadjusted HR 1.75, 95% CI 1.09–2.82). The association was only slightly weaker after adjustment for conventional cardiovascular risk factors, eGFR, proteinuria and hs-CRP.

## Discussion

A risk prediction tool for cardiovascular events and mortality in RTR underestimates risk in some individuals [6–8]. Accurately quantifying risk in this population is therefore challenging. It has been proposed that biomarkers of cardiovascular disease may aid with risk-stratification following kidney transplantation [22].

In this prospective cohort study of 367 RTR, we found a strong independent association between sST2 and adverse patient outcomes including all-cause mortality, cardiovascular mortality and MACE. However, the addition of sST2 concentration to risk prediction models based on clinical risk factors and hs-CRP had little meaningful impact on their predictive accuracy.

Two isoforms of ST2 are of clinical significance: ST2 ligand (ST2L), a transmembrane form, and sST2, a truncated protein which circulates in plasma [23]. The interaction of ST2L with its ligand, IL-33, is cardioprotective, reducing myocardial fibrosis and hypertrophy [24]. sST2 acts as a ‘decoy receptor’ by binding IL-33 and preventing the beneficial effects of its interaction with ST2L [23]. Increased expression of sST2 from cardiomyocytes is induced by mechanical strain, and its concentration correlates with ongoing fibrosis and inflammation [11, 25]. Non-myocardial production of sST2 may also occur, and sST2 has been implicated in the progression of atherosclerotic plaques in animal models [26, 27].

sST2 measurement has been incorporated into clinical guidelines for the purpose of risk-stratifying patients with acute and chronic heart failure [28]. An sST2 concentration > 35 ng/ml is associated with increased risk of mortality in this population [12]. In our study, the association of sST2 with all-cause mortality was significant when the biomarker was treated as a continuous variable and when it was categorised according to this cut-off value. However, the associations with cardiovascular mortality and MACE lost significance when using this cut-off. It is possible these findings represent a loss of statistical power which occurs when continuous variables are dichotomised [29].

Alternatively, the cut-off value validated for use in patients with heart failure may not be applicable to RTR. In a study of patients with ESRD on haemodiafiltration, sST2 concentrations > 35 ng/ml were associated with cardiovascular mortality but the strength of the relationship was greatest when sST2 was dichotomised at a higher cut-off value of 44 ng/ml [16]. Interestingly, in our study, the optimal cut-off values of sST2 concentration differed according to the adverse outcome of interest. For each outcome, the association with sST2 concentration was much stronger when the optimal cut-off values were applied in comparison to the traditional cut-point of 35 ng/ml. This must be interpreted with caution, however, as it recognised that the application of optimal cut-points in the cohort from which they were derived can introduce bias, over-estimating the magnitude of associations leading to overly optimistic estimates of sensitivity and specificity [30].

This study is the first to our knowledge to investigate the prognostic utility of sST2 in RTR, but our findings are consistent with reported results in other patient groups. In a large study of elderly, community-based individuals, elevations of sST2 concentration were associated with heart failure and cardiovascular mortality [31]. Analogous to our results, this study found that the addition of sST2 to existing risk models had only a modest impact on their predictive accuracy. Another study measured sST2 in 200 kidney transplant candidates, 60% of whom were on maintenance dialysis and 40% of whom had CKD 5. sST2 was associated with mortality and cardiovascular events on univariable analysis but did not improve cardiovascular risk prediction in multivariable analysis [32]. This is a common challenge encountered in biomarker research. It has been acknowledged that even when a strong association exists between a biomarker and cardiovascular disease, the addition of the biomarker to risk models often fails to change their C-statistic to a clinically meaningful degree [33].

One challenge when measuring biomarkers in patients with renal disease is the potential for their concentration to be altered by the level of eGFR. In our study, and in others, however, sST2 concentration was not correlated or only weakly inversely correlated with eGFR [14, 15, 34]. A recent study involving 883 patients with CKD and a mean eGFR of 49 ml/min/1.73m^2^ demonstrated an association of elevated sST2 concentration with increased risk of all-cause mortality [15]. The prognostic utility of sST2 in patients with heart failure is also unaffected by reduced eGFR [34]. In a study by Bayes-Genis and colleagues, sST2 improved prediction of adverse outcomes in patients with renal impairment more than in those without [34]. Different pathophysiological pathways may be involved in the development of cardiovascular disease in patients with renal disease. In combination with our study, these findings suggest sST2 may be closely associated with these unidentified pathways in renal impairment.

Our study has several strengths. Its primary strength is the availability of detailed, prospectively collected, follow-up data for all participants who had sST2 concentration measured. The Northern Ireland Kidney Transplant Database provides follow-up data for all RTR from time of transplantation until death. Consequently, the follow-up duration of this study is amongst the longest described in the literature. In addition, our baseline data allowed us to adjust for all traditional cardiovascular risk factors, as well as graft function and proteinuria, in survival models. The C-statistics of these models prior to the addition of sST2 are > 0.75 for all outcomes, suggesting the selected covariates were strongly predictive of adverse outcomes in our cohort. We maximised the precision of sST2 measurement by analysing all plasma samples in duplicate and repeating measurement for samples with an intra-assay CV > 10%.

The limitations of our study also deserve consideration. All recruited RTR were Caucasian, which is reflective of the wider population in Northern Ireland. However, this does limit the generalisability of our results to countries with more racially diverse populations. Participants in this study were recruited between 2000 and 2002, with a significant proportion taking ciclosporin. Therefore, their cardiovascular risk profile may not be equivalent to that of RTR in the modern era. Additionally, echocardiographic reports were unavailable at recruitment and during follow-up. Such reports may have helped elucidate the structural cardiac abnormalities, and therefore the underlying biological pathways, associated with elevated sST2 concentration in RTR. Given that steroid use and proteinuria differed between high and low sST2 groups, residual confounding cannot be fully excluded. Finally, sST2 concentrations were measured only once in each participant, so that the prognostic value of serial sST2 determinations could not be assessed.

## Conclusions

In conclusion, sST2 adds little incremental value to the accuracy of risk prediction models in RTR beyond conventional risk factors and hs-CRP. However, sST2 is associated with mortality and MACE in this population. Further studies are warranted to ascertain the pathobiological pathways associated with elevated sST2 concentration in RTR, and to determine whether these pathways may act as potential therapeutic targets for reduction of cardiovascular risk.

## Supplementary information

**Additional file 1: Figure S1.** A plot of sST2 concentration versus time post-transplant for participants who experienced cardiovascular mortality and those who did not. **Table S1.** Demonstrates the association of sST2 concentration with adverse outcomes in a sex-stratified analysis.

## Data Availability

The datasets used and/or analysed during the current study are available from the corresponding author on reasonable request.

## Abbreviations

BMI: Body mass index
CI: Confidence interval
CKD: Chronic kidney disease
CNI: Calcineurin-inhibitor
CRCRTR: Cardiovascular Risk Calculator for Renal Transplant Recipients
CV: Coefficient of variation
ECG: Electrocardiogram
eGFR: Estimated glomerular filtration rate
ELISA: Enzyme-linked immunoassay
ESRD: End-stage renal disease
HR: Hazard ratio
hs-CRP: High-sensitivity C-reactive protein
IQR: Interquartile range
LVH: Left ventricular hypertrophy
MACE: Major adverse cardiovascular events
MDRD: Modification of Diet in Renal Disease
RTR: Renal transplant recipients
SD: Standard deviation
sST2: Soluble ST2
ST2L: ST2 ligand
