# Supplementary material for: Association of soluble ST2 with all-cause and cardiovascular mortality in renal transplant recipients: a single-centre cohort study
Source: BMC Nephrol. 2020 Jan 28;21:22. doi: 10.1186/s12882-020-1690-6 (PMC6986045; doi:10.1186/s12882-020-1690-6)
Supplement: Supplementary file 1 — Additional file 1: Figure S1. A plot of sST2 concentration versus time post-transplant for participants who experienced cardiovascular mortality and those who did not. Table S1. Demonstrates the association of sST2 concentration with adverse outcomes in a sex-stratified analysis. [file 12882_2020_1690_MOESM1_ESM.docx]

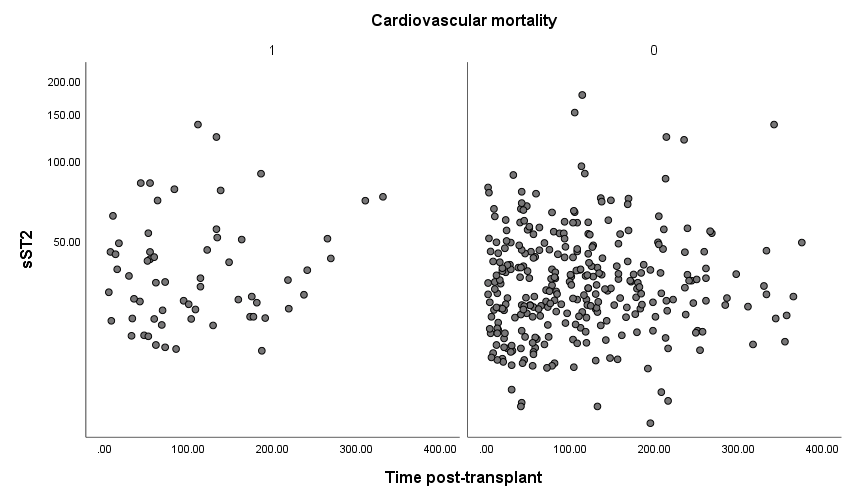


**Figure S1:** Plot of sST2 concentration versus time post-transplant for participants who experienced cardiovascular mortality (denoted 1) and those who did not (denoted 0).

**Table S1:** Association of sST2 concentration with adverse outcomes according to sex.

|  |  | | **Model 1** | | **Model 2** | |
| --- | --- | --- | --- | --- | --- | --- |
|  | **Unadjusted HR**  **(95% CI)** |  | **Adjusted HR (95% CI)** |  | **Adjusted HR (95% CI)** |  |
| **All-cause mortality** |  |  |  |  |  |  |
| sST2 *(per twofold increase)* |  |  |  |  |  |  |
| Total cohort | 1.31 (1.05-1.63) |  | 1.36 (1.05-1.76) |  | 1.33 (1.06-1.67)^a^ |  |
| Male | 1.32 (0.99-1.75) |  | 1.44 (1.02-2.03) |  | 1.32 (0.98-1.78)^a^ |  |
| Female | 1.35 (0.96-1.89) |  | 1.35 (0.90-2.05) |  | 1.47 (1.03-2.11)^a^ |  |
| **Cardiovascular mortality** |  |  |  |  |  |  |
| sST2 *(per twofold increase)* |  |  |  |  |  |  |
| Total cohort | 1.50 (1.05-2.13) |  | 1.65 (1.09-2.48) |  | 1.50 (1.03-2.18)^a^ |  |
| Male | 1.50 (0.97-2.33) |  | 1.67 (0.97-2.88) |  | 1.52 (0.96-2.41)^a^ |  |
| Female | 1.43 (0.77-2.64) |  | 1.86 (0.95-3.63) |  | 1.66 (0.87-3.17)^a^ |  |
| **MACE** |  |  |  |  |  |  |
| sST2 *(per twofold increase)* |  |  |  |  |  |  |
| Total cohort | 1.36 (1.07-1.74) |  | 1.30 (0.97-1.73) |  | 1.40 (1.08-1.80)^b^ |  |
| Male | 1.33 (0.99-1.79) |  | 1.30 (0.92-1.83) |  | 1.32 (0.97-1.79)^b^ |  |
| Female | 1.26 (0.79-2.01) |  | 1.62 (0.92-2.86) |  | 1.40 (0.84-2.34)^b^ |  |

Model 1: adjusted for age, sex, diabetes, history of hypertension, cholesterol/HDL ratio, BMI, smoking status, history of CVD, eGFR, proteinuria category, hs-CRP

^a^Model 2 – mortality: adjusted for age, diabetes, total RRT time (= pre-transplant dialysis time + time-post transplant), serum creatinine, smoking status, history of IHD

^b^Model 2 – MACE: adjusted for age, diabetes, LDL-cholesterol, number of transplant grafts, serum creatinine, smoking status, history of IHD

Abbreviations: BMI = body mass index; CI = confidence interval; CVD = cardiovascular disease; eGFR = estimated glomerular filtration rate; HR = hazard ratio; hs-CRP = high-sensitivity C-reactive protein; IHD = ischemic heart disease; MACE = major adverse cardiovascular events; RRT = renal replacement therapy; sST2 = soluble ST2
